# SMILE, YOU’LL FEEL BETTER: TESTING THE FACIAL FEEDBACK HYPOTHESIS IN YOUNG AND OLDER ADULTS

**DOI:** 10.1093/geroni/igae098.1992

**Published:** 2024-12-31

**Authors:** Nikolina Kravljaca, Jennifer Stanley

**Affiliations:** University of Akron, Akron, Ohio, United States; University of Akron, Akron, Ohio, United States

## Abstract

We investigated age differences in the effect of facial feedback on the emotional experience on a sample of young and older adults. The Facial Feedback Hypothesis proposes that feedback from facial expressions impacts the emotional experience by enhancing or dampening the emotional experience (James, 1884; Darwin 1897). Embodied cognition, the bidirectional relationship between the mind and the body is central to maturational dualism, which suggests older adults show reductions in proprioception, interoception, and physiological reactivity (Niedenthal et al., 2005; Mendes, 2010). Based on this, we expected older adults to show reduced facial feedback effects. Participants were instructed to pose a happy, angry, and neutral facial expression and rate their happiness and anger on a 7-point scale before and during the facial manipulation. We found that in the Angry Condition, there was a main effect of Time, such that both age groups showed reductions in happiness scores, F(1,119) = 86.243, p<.001, η2=.420 and increases in anger, F(1,119) = 29.798, p<.001, η2=.200, as well as a Time x Age Group interaction, F(1,119) = 4.546, p<.035, η2=.03, showing that older adults had a larger decrease in happiness scores, but no age differences in feelings of anger. In the Happy Condition, we did not find any significant age differences in ratings of happiness or anger from pre to post while posing the happy or angry facial expression. Contrary to our hypotheses, these results suggest that older adults do experience facial feedback effects specifically for anger, but not happiness.

